# Apple rootstocks affect functional leaf traits with consequential effects on carbon isotope composition and vegetative vigour

**DOI:** 10.1093/aobpla/plac020

**Published:** 2022-05-05

**Authors:** Erica Casagrande Biasuz, Lee A Kalcsits

**Affiliations:** Department of Horticulture, Washington State University, 1100 North Western Avenue, Wenatchee, WA 98801, USA; WSU Tree Fruit Research and Extension Center, 1100 North Western Avenue, Wenatchee, WA 98801, USA; Department of Horticulture, Washington State University, 1100 North Western Avenue, Wenatchee, WA 98801, USA; WSU Tree Fruit Research and Extension Center, 1100 North Western Avenue, Wenatchee, WA 98801, USA

**Keywords:** Carbon assimilation, carbon isotope composition, dwarfing rootstock, *Malus domestica*, plant water relations, shoot growth

## Abstract

Composite trees combine optimal traits from both the rootstock and the scion. Dwarfing rootstocks are commonly used to reduce shoot vigour and improve fruit quality and productivity. Although growth habits of different rootstocks have been clearly described, the underlying physiological traits affecting scion vigour are not well understood. Plant water status and stem water potential are strongly influenced by water supply and demand through the soil–plant–atmosphere continuum. In the scion, stomata regulate water loss and are essential to prevent hydraulic failure. Stomatal conductance influences leaf carbon isotope composition. Combined, the effects of reduced stomatal conductance and, consequently, carbon fixation may affect tree growth. These differences could also correspond to differences in scion vigour controlled by rootstock genotype. Here, vegetative growth, gas exchange, stem water potential and leaf δ^13^C were compared to determine how rootstocks affect scion water relations and whether these differences correspond to shoot vigour. There was a range in vigour among rootstocks by almost 2-fold. Net leaf carbon assimilation rates were lower in rootstocks with lower vigour. Rootstock vigour was closely associated with leaf gas exchange and stem water potential in the scion and was reflected in leaf δ^13^C signatures. Dwarfing was strongly affected by changes to plant water status induced by rootstock genotype and these changes are distinguishable when measuring leaf and stem δ^13^C composition. These observations indicate that scion water relations and leaf carbon isotope discrimination were affected by rootstock genotype. These results have implications for better understanding dwarfing mechanisms in apple rootstocks and the relationship with water-use traits.

## Teaser

Apple rootstocks are important for controlling scion vigour and strong variation exists in size control among rootstocks. Plant water relations can also affect shoot growth and are strongly influenced by water supply and demand. The effects of rootstock-affected water limitations may reduce gas exchange and, consequently, may also affect tree growth. Here, net leaf carbon assimilation rates and stem water potential were lower in rootstocks with lower vigour. These findings suggest that apple rootstocks affect water movement between roots and the scion which can affect tree vigour through the downstream effects on leaf gas exchange and leaf δ^13^C signatures.

## Introduction

Apples (*Malus domestica*), like many horticultural crops, are clonally propagated and are comprised of a rootstock and a scion. Apple production relies on dwarfing rootstocks which enhance yield efficiency, fruit quality, disease resistance and tolerance to freezing conditions (Mika *et al.* 1981; Palmer and Wertheim 1981; Sansavini *et al.* 1981). In the last 50 years, the adoption of dwarfing rootstocks has transformed apple production because they reduce vigour and increase precocity (Czynczyk 1981; Webster 2001; Marini and Fazio 2018). Currently, there are four size categories for apple rootstocks; dwarfing, semi-dwarfing, semi-vigorous and vigorous (Roper 2001). Despite extensive characterization of rootstock traits in the last century, the mechanisms by which rootstocks control scion growth are still not well defined (Westwood 1970; Jones 1971, 1984).

When plants are grafted, fusing of vascular tissue occurs and can be affected by both rootstock and scion genotype. Dwarfing is the key rootstock-induced trait in apple. Differences in growth among rootstocks can often be seen immediately upon grafting (Roberts 1927) and rootstocks can change the healing of the graft union and stem xylem morphology, both of which can potentially impact scion water relations (Olien and Lakso 1984, 1986; Albacete *et al.* 2015). Several potential mechanisms of dwarfing have been proposed involving hormonal regulation, mechanical limitations or genomic control (Beakbane and Thompson 1940, 1947; Spangelo 1974; Tubbs 1980; Higgs and Jones 1990; Soumelidou *et al.* 1994; Webster 1995; Seleznyova *et al.* 2003; Tworkoski and Miller 2007; Dolgun *et al.* 2009; Gregory *et al.* 2013; Harrison *et al.* 2016). However, phenotypic variation in these traits is difficult to characterize in composite plants. Higher-throughput approaches are needed that capture the effect of rootstocks on scion water relations and downstream effects on plant growth and development.

Changes to stem water potential are normally induced via changes in soil water availability (Suter *et al.* 2019). Stem water potential contributes to the control of stomatal conductance (Buckley 2019). Stomatal behaviour depends on the physiological traits of the plant and the host environment. At night, water is taken up and stored within the plant (Knipfer *et al.* 2019). In the morning, stomata open and transpiration rates increase, reducing leaf water potential and turgor pressure (Dominguez *et al.* 2019). Drought stress forces earlier stomatal closure due to loss of leaf turgor and, consequently, a decline in leaf xylem conductance to avoid excessive negative pressure in xylem (Knipfer *et al.* 2020). A reduction in stomatal conductance is, in part, an effect of a decline in root hydraulic conductance and these effects are mediated partially by responses of stomata to leaf water status (Knipfer *et al.* 2020). Although environmental conditions are key contributors to these responses, limitations in water supply from the soil via intraspecific variation in root traits can also impact stem water potential.

Measurements of stable carbon isotope ratios in plant material can be used to study plant water relations (Saugier *et al.* 2012). Carbon isotope ratios are used to calculate intrinsic water-use efficiency (iWUE) and these ratios can reflect the balance between carbon acquisition and water consumed by transpiration (Cernusak *et al.* 2003; Seibt 2008; Ma *et al.* 2010; Bchir *et al.* 2016). Carbon isotope discrimination occurs during diffusion of CO_2_ to the site of fixation by Rubisco and then, by Rubisco itself. Since carbon discrimination by Rubisco is much greater than that of diffusion and diffusion to the site of fixation by Rubisco is proportional to the ratio of internal CO_2_ concentrations and atmospheric CO_2_ (c_i_/c_a_), less discrimination occurs when stomatal conductance is low (Guy *et al.* 1993). Atmospheric and soil conditions are known to affect iWUE and carbon isotope discrimination in plants (Cernusak 2020). However, in comparative experiments, environmental conditions should be similar for all plants. Differences in stomatal conductance and thus, carbon isotope composition differences should, therefore, be more affected by internal changes in water supply or demand. Vigorous plants, where water supply may be less limited, may have depleted isotopic values compared to water-limited species or cultivars (Lehmann *et al.* 2018). Carbon isotope discrimination can be an effective seasonally integrated proxy for gas exchange and water relations throughout the season (Mills *et al.* 1998). Since these effects on stomatal function and gas exchange are downstream from changes in water relations, the effect that rootstocks have on root water uptake, limitations at the graft union or induced hydraulic limitations in the scion stem or leaves should be clearly reflected in the isotopic signature.

The objective for this study was to assess rootstock-mediated variability in water relations for a common apple scion grafted onto nine rootstocks. We hypothesized that phenotypic differences in vigour among rootstocks will be reflected in their effect on gas exchange and water relations of the scion. Consequently, these effects should have downstream effects on carbon isotope composition of leaves and stems as a carbon source and sink, respectively. These findings will help understand the relationship between water relations and rootstock dwarfing capacity in the scion. Furthermore, clear rootstock-mediated effects on carbon isotope ratios will improve selection efficiency for dwarfing capacity. The knowledge acquired from this study can be applied to better pair rootstocks with specific cultivars, environment conditions, and improve efficiencies for rootstock breeding programs.

## Materials and Methods

### Site description and experimental design

The experiment was conducted using ‘Honeycrisp’ as scion grafted onto nine different rootstocks at Washington State University (WSU) Sunrise Research Orchard in Rock Island, WA, USA (47°18ʹ35.6″N, 120°03ʹ59.5″W). The rootstocks in this experiment were: Budagovsky 9 (B.9), CG.4292, CG.5257, M.9-T337, Geneva 210 (G.210), Geneva 814 (G.814), Geneva 87 (G.87), Geneva 890 (G.890) and Geneva 969 (G.969). The dwarfing capacity of these rootstocks ranged from dwarfing (B.9) to semi-vigorous (G.890). The experiment was planted in 2017 and each rootstock had three replicated plots arranged in a completely randomized design. The soil is a shallow sandy loam soil with good drainage. The orchard was oriented north to south and rows were spaced 3.6 m apart and 0.9 m between trees which were trained to a spindle system and irrigated using at approximately 110 % replacement of estimated evapotranspiration during the growing season using a combination of drip and micro-sprinklers. Trees were managed and pruned according to commercial tree fruit management practices. Experiments took place in 2018 and 2019. In both years, flowers were completely removed from the trees to eliminate any cropping during early growth. Soil volumetric water content and soil temperature were measured using an ECH_2_O 5TM soil moisture and temperature probe (Meter Group, Pullman, WA, USA) (Table 1). Three soil moisture sensors were placed in different locations in the herbicide strip equidistant between two trees at 20 cm depth. Relative humidity and air temperature were measured with a VP-3 sensor (Meter Group, Pullman, WA, USA) placed in the middle row 1.8 m above ground. Each soil probe, air temperature and relative humidity sensors were interfaced with an EM50G data logger (Meter Group, Pullman, WA, USA) and data were logged every 15 min from June to August. Solar radiation and wind speed were obtained through the AgWeatherNet weather stations located immediately adjacent to the WSU-Sunrise Orchard (https://weather.wsu.edu/).

**Table 1.** Mean environmental data from June to August in 2018 and 2019 at Washington State University Sunrise Research Orchard in Wenatchee, WA, USA. Data were recorded from 1 June until 20 August with interval of 30 min between measurements.

	Mean temperature (°C)	Solar radiation (MJ m^−2^)	Relative humidity (%)	Soil temperature (°C)	Soil moisture (m^3^ m^−3^)
2018					
June	19.9	737	41	20.06	0.22
July	26	817	31.6	23.93	0.27
August	23.9	583	38.1	23.06	0.27
2019					
June	21.1	756	37.4	21.07	0.23
July	23.5	728	37.8	23.88	0.19
August	24.5	630	41.3	23.68	0.24

### Physiological measurements

Physiological measurements were made monthly in 2018 and 2019 starting from June through August on days that were cloud-free and with temperatures not exceeding 30 °C. Means were calculated for all sampling times during the season. Maximum stomatal conductance of water vapour (*g*s_w_) and net CO_2_ assimilation were measured on two sun-exposed mature leaves in one tree per plot replicate between 10 am and 12 pm using a LI-6400XT infrared gas analyser (Li-COR, Lincoln, NE, USA). During the experiment the air flow was constant at 400 µmol s^−1^, reference CO_2_ concentration was set at 400 ppm, leaf temperature at 25 °C and photosynthetic photon flux density inside the chamber was set to 1500 μmol m^−2^ s ^−1^. After placing the leaf in the gas exchange chamber, the leaf was allowed to equilibrate until reference and sample values stabilized. Intrinsic water-use efficiency (µmol CO_2_ mol H_2_O^−1^) (Equation (1)) was obtained by calculating the ratio of net photosynthetic rate (µmol CO_2_ m^−2^ s^−1^) to stomatal conductance of water vapour (mol m^−2^ s^−1^).

To assess water status as stem water potential, two fully mature expanded leaves were chosen from inside the canopy closest to the base of the tree and leaf water potential was allowed to equilibrate with the stem in a silver reflective bag for at least 90 min. Then, at solar noon, stem water potential was measured using a Scholander System Pressure Chamber Instrument (PMS Instrument Co., Albany, OR, USA).


$$\begin{array}{l} iWUE=\left(\frac{Net\;\;\;Photosynthetic\;\;\;Rate}{Stomatal\;\;\;Conductance}\right) \end{array}$$ (1)

### Morphological measurements

Morphological measurements took place once a month from June to August in 2018 and 2019.

Current-year shoot growth (cm) was measured from the bud scar that developed at bud break to the apical meristem. For this measurement, six shoots were measured on two trees per replicate. In these same trees, trunk diameter was measured at 10 cm above the graft union and trunk cross-sectional area (TCSA) was calculated.

### Carbon isotope composition (δ^13^C)

The entire portion of new annual growth was excised from three terminal stems per replicate, then leaves and stems were separated for carbon isotope composition analyses. The samples were placed in a paper bag and dried in a chamber with constant air flow. After the samples were completely dry, subsamples of 2 g were taken from shoots and leaves, respectively, and ground to a fine powder for each replicate using a VWR Homogenizer (VWR, Radnor, PA, USA). Once each sample was transformed into fine powder, 3 mg were weighed for leaves and 4 mg were weighed for stems using a high-precision analytical balance (XSE105 DualRange, Metler Toledo, Greifensee, Switzerland) and placed into 5 mm × 9 mm tin capsules (Costech Analystycal Technologies, Inc., Valencia, CA, USA). Prepared capsules were shipped for analysis to the Stable Isotope Core Laboratory at Washington State University. Samples were analysed using an elemental analyser (ECS 4010, Costech Analytical, Valencia, CA, USA) coupled with a continuous flow isotope ratio mass spectrometer (Delta PlusXP, Thermofinnigan, Bremen, Germany). Carbon isotope ratios were determined using the standard Vienna PeeDee belemnite and the values reported in ‘delta’ notations as δ values in permil (‰) (Sharp 2017) as described in Equation (2).


$$\begin{array}{l} \delta^{13}C=\left(\left(\frac{\left(\frac{{}^{13}C}{{}^{12}C}\right)sample}{\left(\frac{{}^{13}C}{{}^{12}C}\right)standard}\right)-1\right)\times 1000 \end{array}$$ (2)

where the ratio of the heavy (^13^C) over the lighter (^12^C) isotope was calculated for the sample and then was divided by the ^13^C/^12^C ratio of the standard.

### Statistical analysis

Composite seasonal means were calculated for net carbon assimilation, stomatal conductance, iWUE and midday stem water potential since there were no interactions between the measurement periods during each season. Data were analysed using a two-way analysis of variance (ANOVA) with rootstock and year as main factors for shoot growth, TCSA, net carbon assimilation, stomatal conductance, iWUE, midday stem water potential, leaf and stem carbon isotope assimilation using PROC GLM (SAS Campus Drive, Cary, NC, USA). Since stem water potential showed an interaction between years, the data were presented for each year, 2018 and 2019 separately. For other data, when no interactions were present, years were pooled together and only the effects of rootstock were presented. Mean separation tests were performed with Tukey’s HSD test (*α* = 0.05). Linear regression was used to test the relationships between individual variables. Figures were prepared using OriginPro 2021 Data Analysis and Graphing Software (OriginLab Corporation, Northampton, MA, USA). Non-linear relationships as well as regression analysis between variables were calculated only for stem and leaf isotope discrimination with SigmaPlot 12.5 (Systat Software Inc., San Jose, CA, USA).

## Results

### Tree growth

**Figure 1.**
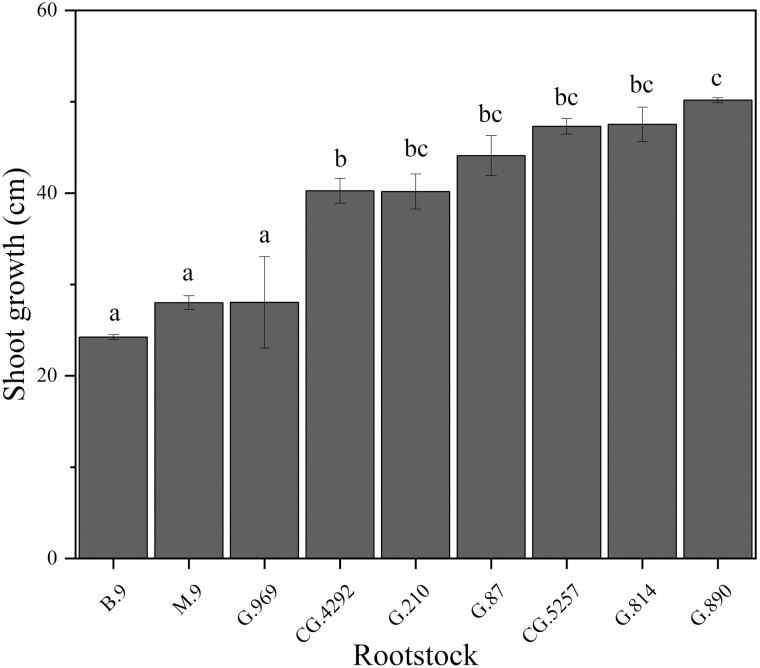
Mean annual shoot growth (cm) of ‘Honeycrisp’ apple trees on B.9, M.9, G.969, CG.4292, G.210, G.87, CG.5257, G.814, G.890 rootstocks. Error bars denote standard error of the mean (*N* = 6). Different letters indicate significant differences among rootstock means determined using a Tukey’s mean separation test (*α* = 0.05).

Rootstock affected scion growth rates that were reflected in both TCSA and shoot growth. One year after planting, TCSA ranged from 2.14 to 6.56 cm^2^ in 2018 and then, 3.55 to 8.90 cm^2^ in 2019 (Table 2). In 2018, TCSA was lower for B.9 among all other rootstocks. In 2019, B.9 had lower TCSA compared to M.9, CG.4292, G.210, CG.5257, G.814, G.890. Rootstock genotype significantly affected terminal shoot growth and annual growth ranged from approximately 20 to 50 cm (Fig. 1). Shoot growth was significantly greater for G.890 compared to B.9, M.9 and G.969. Greater growth was also observed for G.210, G.87, CG.5257 and G.814.

**Table 2.** Mean TCSA of ‘Honeycrisp’ apple trees on B.9, M.9, G.969, CG.4292, G.210, G.87, CG.5257, G.814 and G.890 rootstocks measured in 2018 and 2019. Different letters indicate significant differences among rootstocks means determined using a Tukey’s mean separation test (*α* = 0.05). SEM, standard error of the mean.

Rootstock	2018		2019	
	TCSA (cm^2^)	SEM	TCSA (cm^2^)	SEM
B.9	2.14a	0.10	3.55a	0.29
M.9	5.07d	0.26	8.17b	0.53
G.969	3.80b	0.44	5.81ab	0.28
CG.4292	3.96cd	0.45	7.96b	0.96
G.210	6.56d	0.51	8.19b	0.98
G.87	2.71bc	0.30	6.12ab	0.41
CG.5257	3.72cd	0.47	7.83b	0.93
G.814	4.14d	0.51	8.53b	0.96
G.890	5.15d	0.24	8.90b	0.74

### Scion water relations and leaf gas exchange

**Figure 2.**
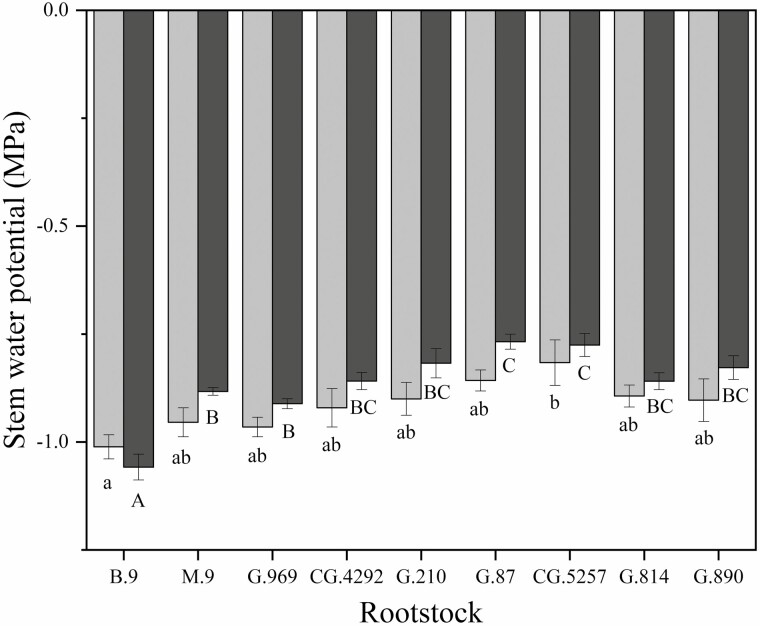
Midday stem water potential (MPa) of ‘Honeycrisp’ apple trees on B.9, M.9, G.969, CG.4292, G.210, G.87, CG.5257, G.814, G.890 rootstocks measured in 2018 (dark grey bars) and 2019 (light grey bars). Error bars denote standard error (*N* = 3). Lowercase letters account for significant differences between rootstocks measured in 2018. Capital letters account for significant differences between rootstocks measured in 2019. Means determined using a Tukey’s mean separation test (*α* = 0.05).

**Figure 3.**
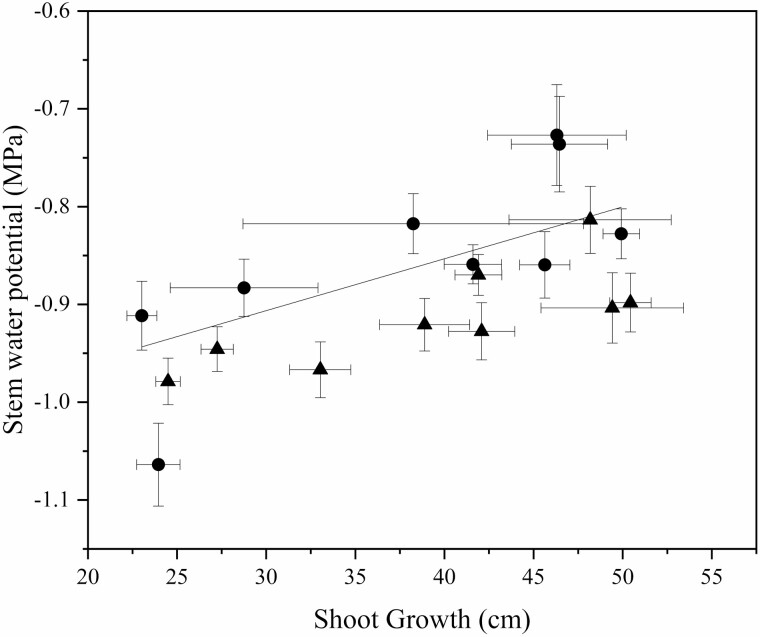
The relationship between stem water potential (MPa) and shoot growth (cm) for 2018 (triangles) compared to measurements from 2019 (circles) (*P* < 0.01, *r* = 0.678) for ‘Honeycrisp’ apple trees grafted on nine rootstock genotypes measured in 2018 and 2019. The lines indicate the best-fit adjusted linear regression for the combined data points.

**Figure 4.**
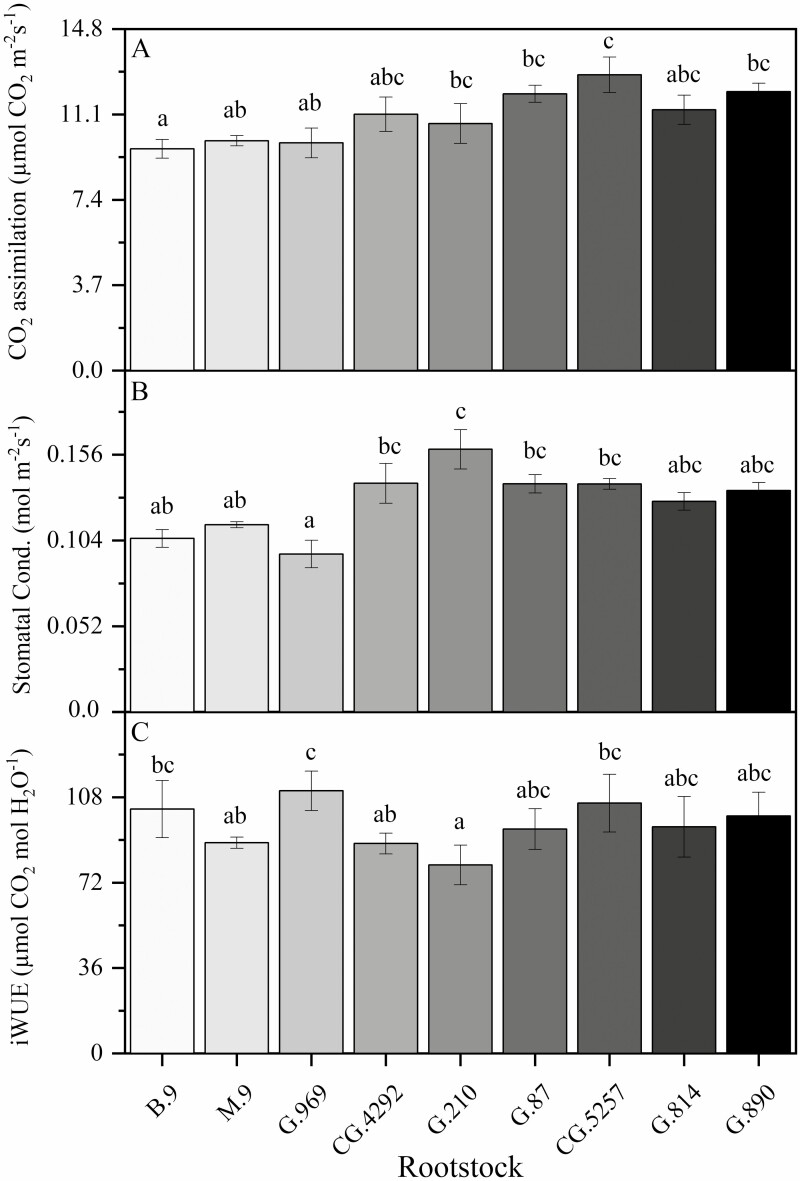
(A) Net CO_2_ assimilation (µmol CO_2_ m^−2^ s^−1^); (B) stomatal conductance (mol m^−2^ s^−1^); and (C) iWUE (µmol CO_2_ mol H_2_O^−1^) for ‘Honeycrisp’ apple trees on B.9, M.9, G.969, CG.4292, G.210, G.87, CG.5257, G.814, G.890 rootstocks measured in 2018. Error bars denote standard error (*N* = 6). Different letters indicate significant differences among rootstock means determined using a Tukey’s mean separation test (*α* = 0.05).

**Figure 5.**
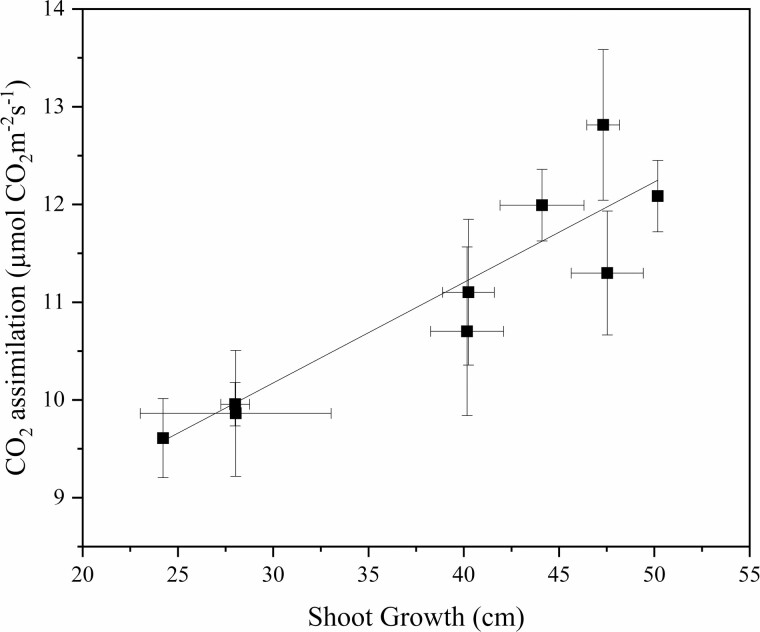
The relationship between mean net CO_2_ assimilation (µmol CO_2_ m^−2^ s^−1^) and annual shoot growth (cm) (*P* < 0.001, *r* = 0.923) for ‘Honeycrisp’ apple trees on nine rootstock genotypes. Each data point represents the mean for both years for each rootstock. The lines indicate the best-fit linear relationship for the combined data points.

**Figure 6.**
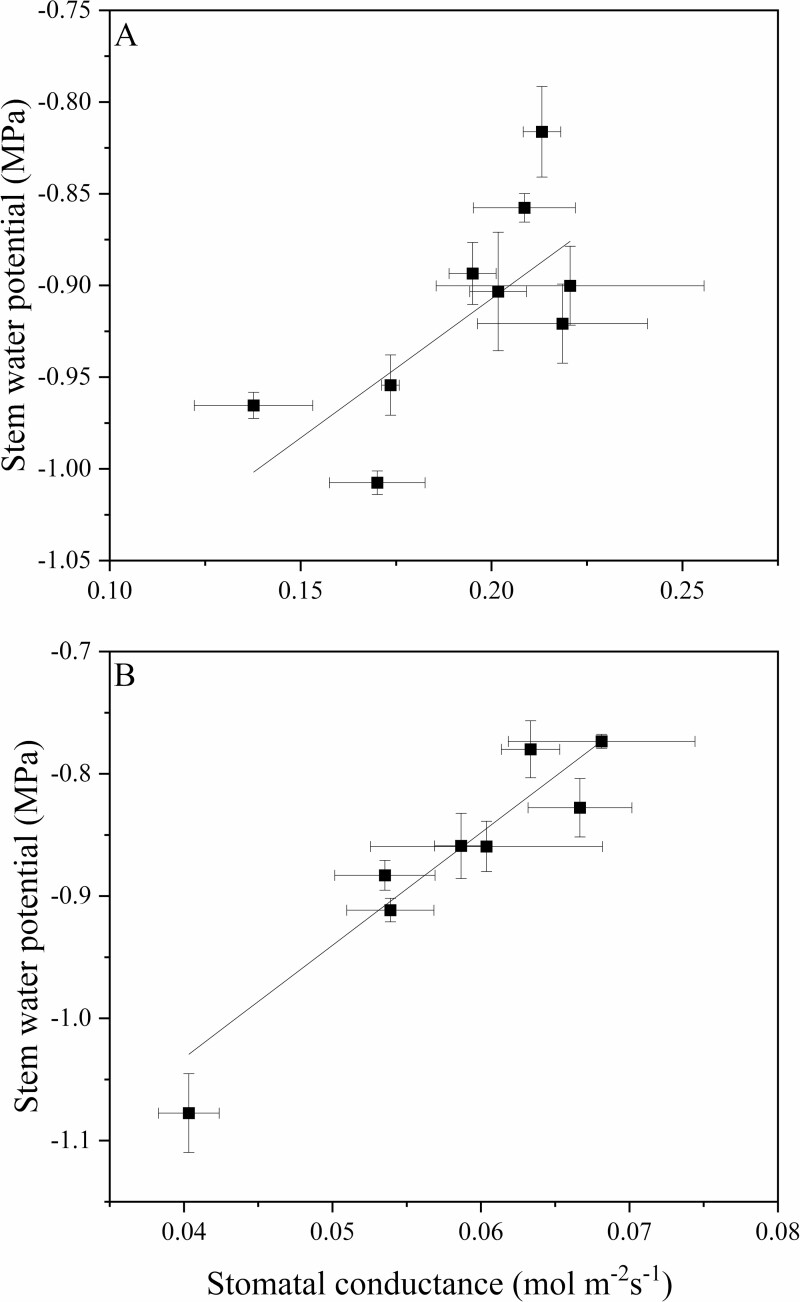
(A) The relationship stem water potential (MPa) and stomatal conductance (mol m^−2^ s^−1^) in 2018 (*P* < 0.05, *r* = −0.708) and (B) the relationship between stem water potential (MPa) and stomatal conductance 2019 (*P* < 0.001, *r* = 0.974) for ‘Honeycrisp’ apple trees on nine rootstock genotypes. The line indicates the adjusted linear regression for the combined data points.

Rootstock affected midday stem water potential in both years (Fig. 2). In this irrigated orchard, mean midday stem water potential (Ψ_m_) did not range beyond −0.8 to −1.0 MPa. B.9 had the lowest stem water potential of all rootstock genotypes. However, in 2018, Ψ_m_ for B.9 was only significantly different from CG.5257. In 2019, differences among rootstocks were greater but trends were similar to 2018. Ψ_m_ was significantly lower for B.9 than more vigorous rootstocks like G.87, CG.5257 and G.890. Midday stem water potential was strongly correlated with seasonal shoot growth (Fig. 3) indicating that rootstocks that confer higher vigour in the scion also contribute to greater stem water potential. Leaf gas exchange traits measured mid-morning differed less among rootstocks than shoot growth or trunk diameter. Net carbon assimilation was the lowest for B.9 at approximately 9 µmol CO2 m^−2^ s^−1^ and the highest for CG.5257 at approximately 13.5 µmol CO_2_ m^−2^ s^−1^ (Fig. 4). There was also a significant relationship between net carbon assimilation and seasonal shoot growth (*r* = 0.923; *P* < 0.001) (Fig. 5). Stomatal conductance ranged from 0.10 to 0.19 mol m^−2^ s^−1^ among rootstocks (Fig. 4). G.969 presented the lower stomatal conductance values and was significantly different from G.4292, G.210, G.87 and CG.5257. Intrinsic water-use efficiency was lowest for G.210 but was higher for G.969 (Fig. 4). Midday stem water potential also strongly correlated with stomatal conductance on both years, 2018 and 2019, respectively (Fig. 6). The strong positive relationship between rootstock-mediated vegetative vigour and water relations indicated that rootstocks limiting water and maintaining a lower inherent stem water potential will also have less vigour.

### Carbon isotope composition

**Figure 7.**
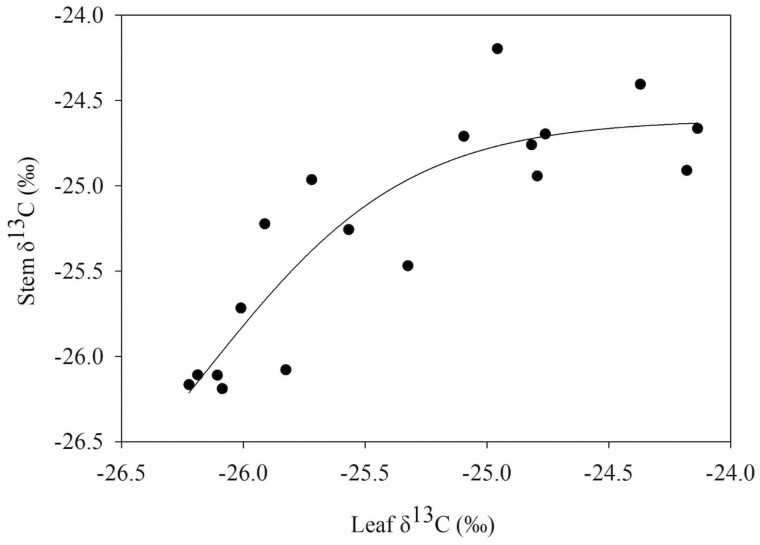
The relationship between leaf δ^13^C (‰) isotope and stem δ^13^C isotope (‰) for ‘Honeycrisp’ apple trees on nine rootstock genotypes (*P* = 0.05, *r* = 0.8914). Each data point represents the mean for both years for each rootstock. The line indicates the best-fit adjusted non-linear regression for the combined data points.

**Figure 8.**
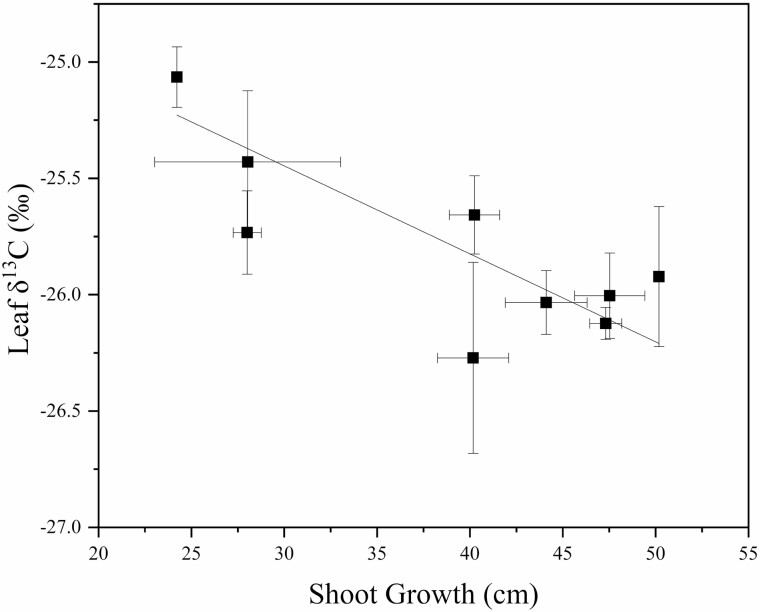
The relationship between mean leaf δ^13^C isotope (‰) composition and shoot growth (cm) (*P* < 0.001, *r* = −0.924) for ‘Honeycrisp’ apple trees on nine rootstock genotypes. Each data point represents the mean for both years for each rootstock. The lines indicate the adjusted linear regression for the combined data points.

**Figure 9.**
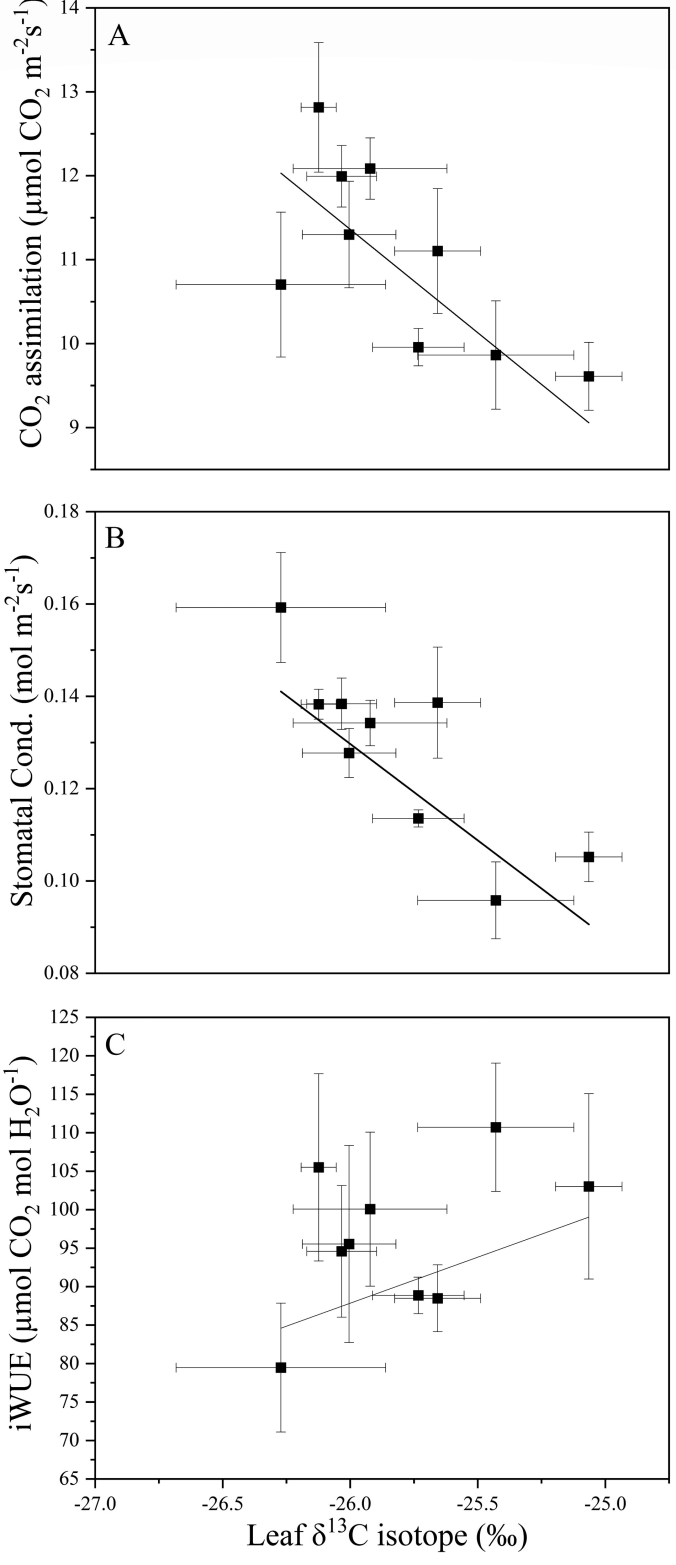
(A) The relationship between CO_2_ assimilation (µmol CO_2_ m^−2^ s^−1^) and leaf δ^13^C isotope (‰) (*P* < 0.01, *r* = −0.873); (B) the relationship between stomatal conductance (mol m^−2^ s^−1^) and leaf δ^13^C isotope (‰) (*P* < 0.01, *r* = −0.807); (C) the relationship between iWUE (µmol CO_2_ mol H_2_O^−1^) and leaf δ^13^C isotope (‰) (*P* > 0.05, *r* = 0.373) for ‘Honeycrisp’ apple trees on nine rootstock genotypes. Each data point represents the mean for both years for each rootstock. The lines indicate the adjusted linear regression for the combined data points.

**Figure 10.**
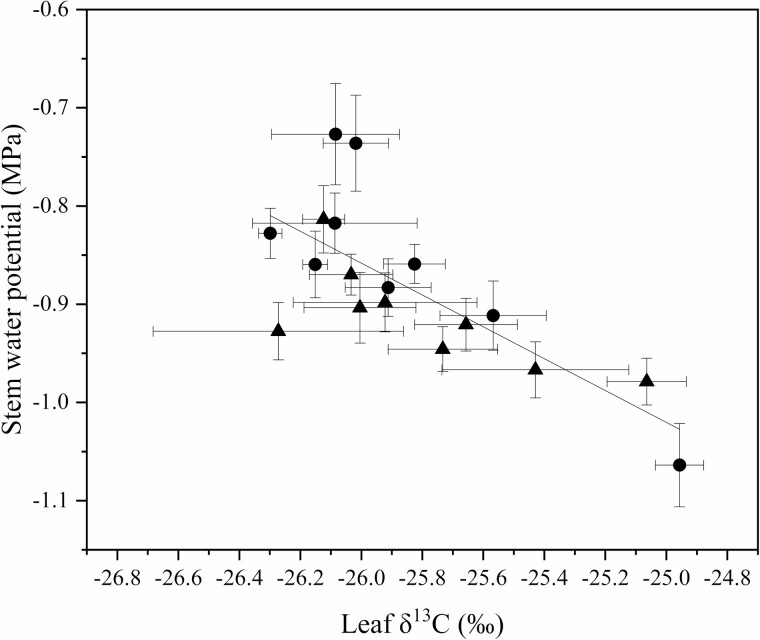
The relationship stem water potential (MPa) and leaf δ^13^C isotope (‰) in 2018 (triangles) and 2019 (circles) for ‘Honeycrisp’ apple trees on nine rootstock genotypes (*P* < 0.001, *r* = −0.906). The line indicates the adjusted linear regression for the combined data points.

Rootstock affected leaf and terminal stem carbon isotope composition (Table 3). In 2018, the rootstocks M.9 and G.969 had less depleted values for leaves when compared to CG.5257. Leaf δ^13^C for all rootstocks was approximately −25 ‰ and ranged by approximately 1 ‰ among rootstock genotypes for both 2018 and 2019. Stem δ^13^C values were closer to −24.5 ‰ and ranged by approximately 2 ‰. At the stem level, although there were no statistical differences, δ^13^C for B.9 was the most enriched and δ^13^C for G.210 was the most depleted. In 2019, for stems, B.9 was more enriched than G.210, G.87, CG.5257, G.814 and G.890. There was a positive non-linear relationship between δ^13^C in leaves and stems (*P* = 0.05, *r*^2^ = 0.8914) (Fig. 7). The data demonstrate that the carbon isotope composition of non-photosynthetic tissues from stem tissue is more enriched compared with leaves of the same branch but converge when values are both on the high or low end of the range in variation observed in this experiment. Carbon isotope composition in leaves was also strongly and negatively correlated with seasonal shoot growth (*P* < 0.001, *r* = −0.924) (Fig. 8). Rootstocks with lower vegetative vigour had higher δ^13^C in both stems and leaves. Net CO_2_ assimilation and stomatal conductance correlated strongly and negatively with δ^13^C (*P* < 0.01, *r* = −0.873; *P* < 0.01, *r* = −0.807) (Fig. 9). As expected, this effect was even more evident for more vigorous rootstocks. Successively, rootstock-mediated variability in stem water potential were negatively related to leaf δ^13^C (*P* < 0.001, *r* = −0.906) (Fig. 10).

**Table 3.** Mean stem and leaf carbon isotope composition (‰) for 2018 and 2019 of ‘Honeycrisp’ apple trees on B.9, M.9, G.969, CG.4292, G.210, G.87, CG.5257, G.814, G.890 rootstocks measured in 2018 and 2019. Different letters indicate significant differences among rootstock means determined using a Tukey’s mean separation test (*α* = 0.05). SEM, standard error of the mean.

Rootstock	2018				2019			
	Leaf δ^13^C (‰)	SEM	Stem δ^13^C (‰)	SEM	Leaf δ^13^C (‰)	SEM	Stem δ^13^C (‰)	SEM
B.9	−24.37ab	0.30	−24.41a	0.04	−24.62a	0.09	−23.67a	0.32
M.9	−24.18a	0.13	−24.91a	0.05	−25.53bc	0.26	−24.81ab	0.10
G.969	−24.14a	0.44	−24.67a	0.17	−25.02abc	0.46	−24.30ab	0.42
CG.4292	−24.82ab	0.26	−24.76a	0.17	−25.09abc	0.16	−25.27ab	0.17
G.210	−25.33ab	1.20	−25.47a	0.34	−25.04abc	1.18	−25.25b	0.4
G.87	−25.10ab	0.36	−24.71a	0.21	−25.66c	0.03	−25.2b	0.16
CG.5257	−25.72b	1.07	−24.97a	0.08	−25.40bc	0.47	−25.44b	0.04
G.814	−24.79ab	0.19	−24.94a	0.11	−25.54bc	0.12	−25.45b	0.19
G.890	−24.76ab	0.06	−24.70a	0.07	−24.92ab	0.16	−25.36b	0.17

## Discussion

The influence of rootstocks–scion water supply and demand dynamics are important for understanding the influence of rootstocks on scion growth and development (Higgs and Jones 1990). Here, under water replete conditions, rootstocks affected stem water potential, leaf gas exchange and carbon isotope composition in both leaves and stems also impacted in the dwarfing capacity of the scion. Rootstocks with a high dwarfing capacity such as B.9 had lower stem water potential, net CO_2_ assimilation, stomatal conductance than other, more vigorous rootstocks. Rootstocks with lower stem water potential also showed more strongly restricted growth and trunk diameter.

In apple, stem water potential has been reported to vary between −0.8 and −1.0 MPa in fully irrigated trees (Naor and Cohen 2003). Here, stem water potential measurements corresponded with previously reported values. Similar to Valverdi *et al.* (2019) and Valverdi and Kalcsits (2021), more dwarfing rootstocks increased inherent water limitations indicated by lower stem water potential, stomatal conductance and transpiration. This was especially true in this study as trees matured from 2018 to 2019. Rooting volume may, in part, contribute to some of these responses (Costes and Garcia-Villanueva 2007; Ma *et al.* 2013; Harrison *et al.* 2016; Foster *et al.* 2017). However, in the semi-arid environment where this study occurred, more than 95 % of the water was supplied through drip irrigation and with a young orchard, irrigated soil volume was similar for all rootstocks. Therefore, differences observed among rootstocks were more likely to result from limitations in water uptake and transport to the scion rather than simply access to water within the soil profile.

The degree of scion dwarfing by rootstocks affected water relations and leaf physiology. These rootstock-induced changes in leaf function also translated to differences in carbon isotope composition in both leaves and stems. In this study, rootstocks modulated scion leaf water relations by affecting stomatal conductance and leaf gas exchange which had downstream effects on δ^13^C composition. There have been previous studies which have reported differences in water relations when different rootstocks were used in woody plants (Bongi *et al.* 1994; Padgett-Johnson *et al.* 2000; Gibberd *et al.* 2001; Cohen and Naor 2002; Ma *et al.* 2010; Liu *et al.* 2012a; Galbignani *et al.* 2016; Peccoux *et al.* 2018; Villalobos-Gonzales *et al.* 2019; Frioni *et al.* 2020). However, these studies did not show the linear relationship between these traits among many phenotypically diverse rootstocks. More often, differences were reported among scion cultivars or among treatments that affect water supply to the roots. Liu *et al.* (2012b) described how rootstocks manipulated stomatal conductance and photosynthetic capacity in ‘Gale Gala’. Here, carbon isotope composition was different for ‘Honeycrisp’ when grafted to apple rootstocks with differences in dwarfing capacities. A reduction in discrimination against ^13^CO_2_ may indicate an increase in water-use efficiency (Jones 1984; Farquhar *et al.* 1989). Differences in carbon isotope composition were not consistent with measured differences in water-use efficiency; carbon isotope composition integrates gas exchange over a much longer period, thus not necessarily surprising to see such inconsistency.

In apples, carbon isotope composition was reported to be lower for Fuji compared to Braeburn apples because of limitations in stomatal and leaf respiration rates (Massonnet *et al.* 2007). In this study, less-negative δ^13^C for more dwarfing rootstocks compared to more vigorous rootstocks indicates increased conservation of water through lower stomatal conductance in leaves, but was not related to iWUE. These effects on water relations had downward effects on scion vigour and were consistent with our knowledge of dwarfing capacity of the rootstocks tested in this study (Seleznyova *et al.* 2008; Kosina 2010; Tworkoski and Fazio 2015; Foster *et al.* 2017).

The positive relationship between carbon isotope composition between leaf and stem demonstrates short-term and long-term impacts of rootstocks on scion water relations and carbon assimilation. We observed enrichment of stems compared to leaves and this is well aligned with previous studies (Arndt and Wanek 2002; Cernusak *et al.* 2005). Specifically, B.9 showed a clear divergence in carbon isotope composition between stem and leaves where leaves were depleted relative to stems. Cernusak *et al.* (2009) presented six hypotheses for this effect. One of the outlined hypotheses to explain enrichment of non-photosynthetic tissues compared with their leaves rely on post-photosynthetic carbon fractionation due to seasonal separation of growth (Cernusak *et al.* 2009). Development and growth of leaves occur in spring with plentiful soil water content. Since variability in carbon discrimination is mainly determined by the ratio of intercellular to ambient CO_2_ partial pressure, the photosynthetic carbon assimilation is heavily discriminating against ^13^C at this time (Werner and Gessler 2011). Wood growth occurs later in the season, when temperatures and vapor pressure deficit (VPD) are higher which can reduce stomatal conductance producing less discrimination against ^13^C at the time carbon is used for synthesis of non-photosynthetic tissues (Cernusak *et al.* 2009). Differences in δ^13^C between leaves and stems were less pronounced when discrimination and vegetative vigour were also greater. Since more vigorous rootstocks extend growth into later parts of the season, we could expect a greater agreement in δ^13^C between leaves and stems than for less vigorous rootstocks like B.9.

The most obvious influence of dwarfing rootstock is the ability to produce lower vegetative biomass relative to more vigorous rootstocks. Trunk diameter and shoot extension measurements are two of the main traits used to characterize tree growth (Lauri *et al.* 2006). In both years, B.9 produced the smallest scion compared to all other rootstocks and our observations were similar to Gjamovski and Kiprijanovski (2011). Corresponding reductions in stem water potential, an accurate measure of plant water status (Shackel *et al.* 1997), were observed among rootstock cultivars.

The rootstock-modulated effect on scion water relations represents an important component in the understanding of rootstock–scion water use (Higgs and Jones 1990). When net carbon assimilation was measured among rootstocks, we observed that less conservative rootstocks like semi-dwarfing G.890 had higher photosynthetic rates. These results follow similar patterns with those reported by Fallahi *et al.* (2001), who reported lower net photosynthetic rates for B.9 compared to more vigorous rootstocks like Ottawa 3 and M.7. Clearly, the capacity of the tree to improve net carbon assimilation and thus, increase carbon fixation is related to the ability of the rootstock to supply water to the leaves (Koepke and Dhingra 2013). Therefore, the hypothesis that reduced growth of dwarfing rootstocks may be associated with rootstock water restrictions at a particular point in the tree was supported by this study as has been suggested by past research (Atkinson and Else 2001; Cohen and Naor 2002; Atkinson *et al.* 2003; Cohen *et al.* 2003; Cohen *et al.* 2007; Tworkoski and Fazio 2015). These restrictions could either occur at the root or the graft union, or in the impact of rootstocks on stem and leaf hydraulic traits. We acknowledge that this is not completely addressed by this study and more work is needed to better discern how rootstocks might affect each point of resistance within the plant.

In conclusion, we report the effect of rootstocks on carbon isotope composition in leaves and stems and show the close association between leaf gas exchange and stem water potential. Rootstocks were able to strongly affect water-use efficiency and carbon assimilation for the same apple scion. Furthermore, there was a close association between rootstock-influenced gas exchange and overall shoot vigour under uniform soil moisture conditions. Rootstocks that were more vigorous have more negative carbon isotope composition indicating lower water restrictions in transport to above-ground parts compared to rootstocks with lower vigour. While there have been numerous studies comparing water relations for dwarfing and non-dwarfing apple rootstocks, this is the first time that scion water relations were associated with carbon isotope discrimination across a range of dwarfing rootstocks. Finally, these results increase our understanding of the physiological mechanisms underlying dwarfing in composite woody plants like apple and show how below-ground traits imparted from rootstocks can affect water-use efficiency and carbon isotope composition in leaves and stems.

## Data Availability

Please contact the author for data requests, and the data will be made available upon your request.

## Notes

**Form & Function. Chief Editor:** Kate McCulloh
